# Kinematics of gray whale (*Eschrichtius robustus*) foraging on infaunal ghost shrimp (*Neotrypaea californiensis*)

**DOI:** 10.1242/jeb.252034

**Published:** 2026-09-18

**Authors:** Hannah M. Clayton, Dave E. Cade, James A. Fahlbusch, Jessica Kendall-Bar, John Calambokidis, Nicholas D. Pyenson, Jeremy A. Goldbogen

**Affiliations:** ^1^ Oceans Department, Doerr School of Sustainability, Stanford University, CA 94305, USA; ^2^ Cascadia Research Collective, 218 1/2 W 4th Ave., Olympia, WA 98501, USA; ^3^ Scripps Institution of Oceanography, University of California San Diego, 8622 Kennel Way, La Jolla, CA 92037, USA; ^4^ Department of Paleobiology, National Museum of Natural History, Smithsonian Institution, Washington, DC 20560, USA

**Keywords:** Integrative, Kinematics, Foraging, 3D model

## Abstract

Emergent technologies and integrative approaches have transformed our ability to understand organismal behavior, physiology and evolutionary ecology. Baleen whales (Mysticeti) have evolved the largest body sizes by filter feeding on dense aggregations of prey in diverse ocean ecosystems. Although the wide range of foraging modes and prey capture abilities among mysticetes has been well documented, the functional morphology underlying these relationships remains poorly understood. Gray whale (*Eschrichtius robustus*) morphology and ecological niche contrast starkly with those of closely related lunge-feeding rorqual species (Balaenopteridae), with gray whales foraging closer to the coastline using longer skulls and flatter mandibles in comparison to most rorquals. We used video and motion-sensing biologging tags to quantify the kinematics of gray whales feeding on infaunal ghost shrimp (*Neotrypaea californiensis*) in the shallow waters of the northern Puget Sound, Washington, USA. Tag-based kinematics were integrated with 3D models and morphological dimensions of the skull to calculate the skull area in contact with the seafloor during each feeding roll, resulting in an average minimum benthic contact area of 4.9±1.1 m^2^. Daily rates of feeding events (mean±s.d. 292±153) were comparable to those of lunge-feeding rorqual species despite using an average of 47.3% (weighted by deployment length) of the available high-tide feeding window. We posit that the paddle-like mandibles of gray whales reflect an adaptation for infaunal prey capture that may facilitate benthic suction filter feeding in shallow coastal habitats, potentially opening a unique coastal foraging niche among mysticetes.

## Teaser

**Summary:** Integration of biologging data and 3D models to estimate feeding rates and spatial foraging characteristics for gray whales suggests their paddle-like mandibles are an adaptation for benthic suction filter feeding in shallow coastal habitats.

## INTRODUCTION

The melding of form and function in comparative biomechanics over the last few decades has proven a key link in understanding organismal biodiversity, evolution and variation in fitness at multiple scales (Blob et al., 2023). The form, function and behavior of organisms are driven, in large part, by the acquisition and use of energy. Understanding how animals forage successfully can yield insight into their physiology, ecology and the evolution of their energetics, as well as potential adaptive limits under environmental change (Costa et al., 2004; Pulliam and Pyke, 2008; Meester et al., 2018). Marine organisms exhibit spectacularly diverse foraging strategies, perhaps in response to the diffusion of energy across far larger and complex food webs relative to those in the terrestrial domain (Vogel, 1996; Benoit-Bird, 2023). Although average food densities in the ocean are low, seasonal resource pulses driven by dynamic ocean currents create foraging hot spots with dense prey patches (Nemoto, 1970; Benoit-Bird et al., 2013; Abrahms et al., 2019).

**Fig. 1.**
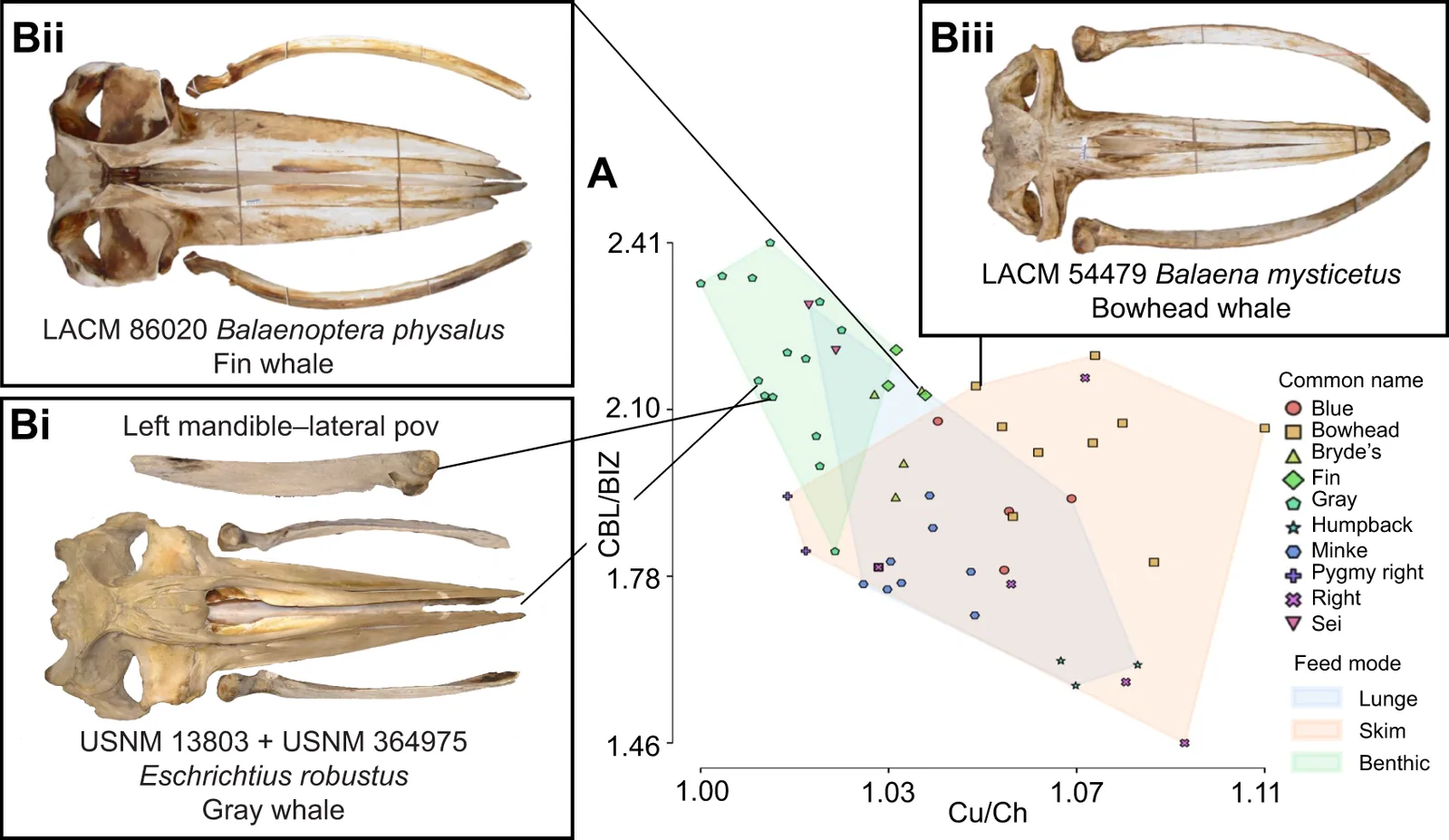
**Cranial and mandibular morphology in skim, lunge and benthically feeding baleen whale species.** (A) Mandible length measurements from museum specimens: the ratio of condylobasal length (CBL; skull length) to bizygomatic width (BIZ; skull width) is represented on the *y*-axis (CBL/BIZ) while the ratio of lower mandible curved length (Cu) to its chord length (Ch) is represented on the *x*-axis (Cu/Ch). (B) Images of dorsal mandibular and cranial morphology of (i) adult gray whales (mandibles and skull belong to two different specimens), (ii) an adult fin whale and (iii) an adult bowhead whale included in the measured specimens in this study.

Baleen whales (or mysticetes) are cetaceans that evolved a wide range of filter-feeding adaptations to exploit these types of ephemeral prey patches at predictably high levels of energetic efficiency (Goldbogen et al., 2019). They use several different modes of filter feeding, ranging from low-speed continuous ram feeding (balaenids) to highly acrobatic lunge-feeding maneuvers (rorquals), and each strategy is associated with unique cranial morphologies (Werth, 2004). Gray whales (*Eschrichtius robustus*) are predominately suction filter feeders that forage on benthic crustaceans in Arctic continental shelves (Nerini*,* 1984), but are also capable of feeding on a wide variety of prey not just in the benthos. Recent and historical observations coupled with modern molecular analyses suggest high prey selection plasticity across habitats (Webber et al., 2024; Gelippi et al., 2022; Frasier et al., 2011; Lagerquist et al., 2019; Lang et al., 2014; Moore et al., 2007; Dunham and Duffus, 2001; Nerini, 1984), which may have enabled several different feeding modes in diverse environments throughout their evolution (Pyenson and Lindberg, 2011). Unlike balaenids and rorquals, both of which exhibit strongly curved lower mandibles (Pyenson et al., 2013), gray whales possess flat paddle-like mandibles (Fig. 1Bi; Table S1) that may allow a large contact area with the seafloor. Although both the rorqual and balaenid cranial structure has been shown to be composed of a unique suite of morphological adaptations which enhance the volumetric capacity of the oropharyngeal cavity and therefore feeding performance (Werth, 2004; Goldbogen et al., 2010, 2017; Werth and Potvin, 2024), we understand far less about how gray whale cranial morphology may relate to feeding efficiency. The filter-feeding morphology of gray whales is hypothesized to enhance benthic feeding in near-shore environments (Nerini, 1984; Alter et al, 2007; Pyenson et al., 2013), but there are also reports of gray whales foraging above the seafloor (Gilmore, 1961; Sund, 1975) and at the sea surface (Norris et al., 1977; Webber et al., 2024) using a diverse range of behavioral strategies. Gray whales have been reported to target over 80 different species of prey (Nerini, 1984), ranging from swarms of mysid shrimp along the Pacific coastline of Oregon and Washington State (Hildebrand et al., 2021, 2022; Colson et al., 2024) to anchovy in San Francisco Bay, California (Webber et al., 2024). Collectively, these studies suggest that gray whales are simultaneously prey generalists and coastal foraging specialists, perhaps reflecting an evolutionary history associated with environmental change along coastal ecosystems that the lineage *Eschrichtius* has encountered over the past ∼2.6 Ma (Pyenson and Lindberg, 2011). Two distinct sub-populations of gray whales, the Eastern North Pacific and Western North Pacific subpopulations, are recognized today (Carretta et al., 2023). Eastern North Pacific gray whales are largely heralded as a conservation success story given their numbers rebounded from a few thousand to an estimated ∼20,000 individuals following the cessation of whaling in the 1980s. This recovery may, in part, be due to their remarkable foraging plasticity and ability to find and exploit novel prey environments that may not be easily accessible to other baleen whale species (Pyenson and Lindberg, 2011).

Recently, smaller subgroups of the Eastern North Pacific population have been recognized based on their atypical foraging and migratory ecology. The Pacific Coast Feeding Group is a small (*n*=213) group of Eastern North Pacific gray whales that forage along the coastline of Oregon and Washington State, USA, and up to southern British Columbia, Canada, in lieu of migrating to traditional foraging grounds in the Arctic Bering and Chukchi Seas each year (Calambokidis et al., 2002; Harris et al., 2025), and they are thought to be matrilineally related (Calambokidis et al., 2002; Lang et al., 2014). Even smaller in number (*n*≈24) are the ‘Sounders’ (hereafter NPS gray whales), a group of unrelated individuals that perform migratory stopovers each spring to feed on ghost shrimp in the northern Puget Sound of Washington State (Weitkamp et al., 1992; Calambokidis et al., 2024). While most NPS gray whales presumably continue their migration northward, a couple individuals have been known to stay year-round. These subgroups raise questions concerning the transmission of foraging information across generations, how energetics may relate to migratory ecology, and the proliferation of gray whales' adaptive capacity throughout their evolutionary history, especially when compared with other mysticete populations, some of which exhibit a narrower range of prey preferences and have recovered poorly post-whaling.

Most of our understanding of gray whale foraging has come from surface-based observations speculating on feeding behavior at depth based on sediment plumes at the surface. Starting in the 1980s, researchers used side-scan sonar and surface-based observations in the Bering Sea (Nerini, 1984) and elsewhere throughout their range (Cacchione et al., 1987; Oliver et al., 1984; Kvitek and Oliver, 1985; Woodward and Winn, 2006) to deduce that gray whales created pits in the seafloor while feeding. These studies primarily concerned Eastern North Pacific gray whales foraging on amphipods (*Ampelisca* spp.) on the continental shelf of the Bering Sea, and showed that their feeding mobilized sediment turnover and provided foraging opportunities for several species of marine seabirds (Harrison, 1979; Oliver et al., 1984; Anderson and Lovvorn, 2008; Hazen et al., 2019; Moore, 2008; Moore et al., 2022). Two decades later, digital motion-sensing recorders (DTAGS) deployed on a small set of gray whales foraging along the coast of British Columbia, Canada, revealed discrete lateralized rolling events, when a whale rolled to its right side to presumably suck in both prey and sediment from the benthos (Woodward and Winn, 2006). Recently, researchers have continued biologging research, often paired with secondary data streams such as drone-based observations and theodolite tracking, to explore the influence of prey dynamics on foraging behavior, energetics and ecology in the Pacific Coast Feeding Group (Hildebrand et al., 2021, 2022; Colson et al., 2024) and to understand fine-scale differences in foraging tactic use relative to body condition and ontogeny (Bird et al., 2024a,b; Bird et al., 2025). However, we still lack even foundational performance metrics (e.g. kinematics, feeding rates) needed to understand foraging energetics and form–function relationships in this enigmatic and monotypic lineage, in part due to logistical difficulties in quantifying these domains in such an inaccessible and cryptic species.

**Fig. 2.**
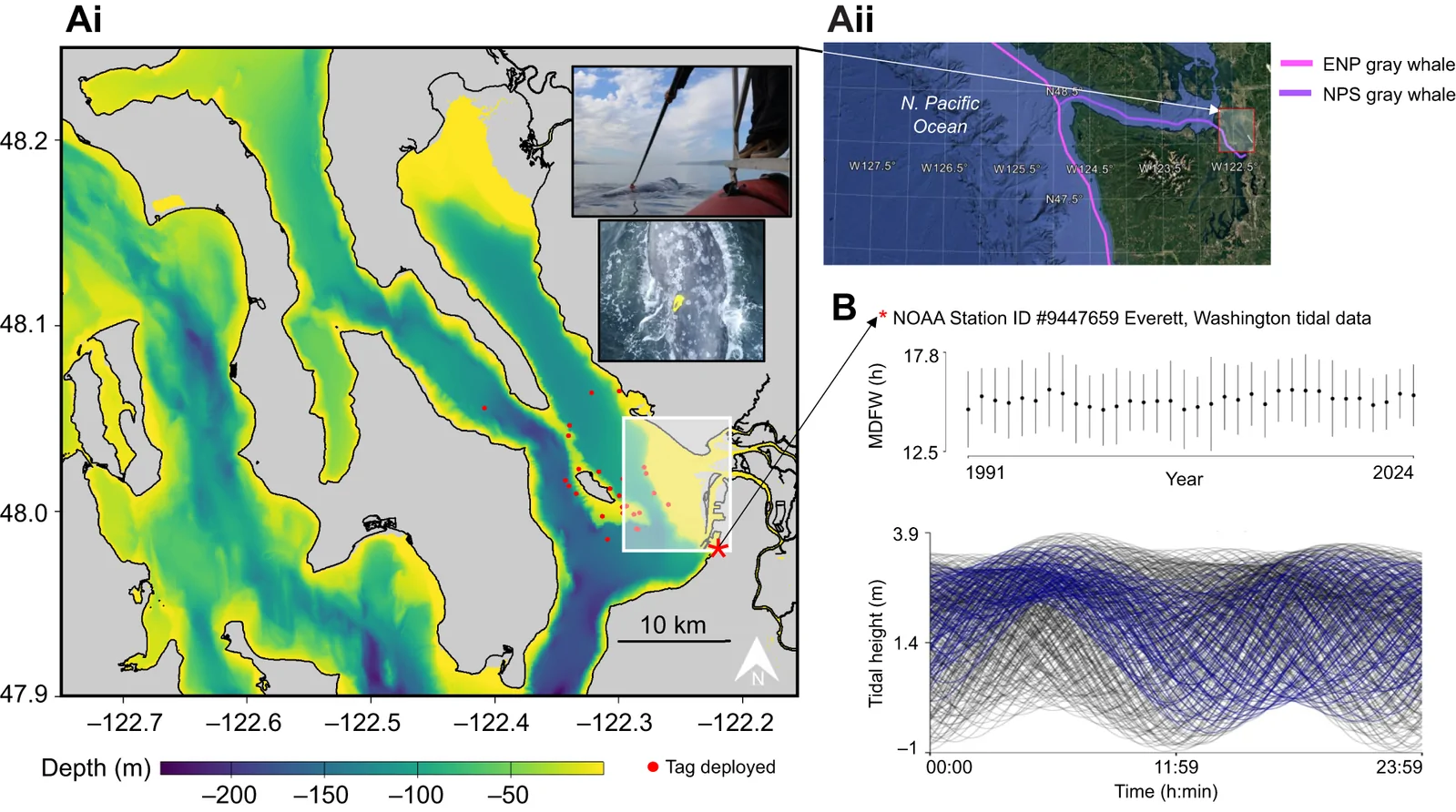
**Description of the study area.** (Ai) Digital surface elevation model data overlaid onto a map of the study area. Red circles indicate where tags were deployed on gray whales for this study since 2015. Inset images overlaid on the map show the two deployment methods used: pole (top) and drone based (bottom). The gray shaded box highlights the Snohomish Delta, a key prey habitat. The red asterisk shows the location of the NOAA tidal station from which plot B data originated. (Aii) A zoomed out view of the region with the study area highlighted by the red box. The pink lines indicate a rough approximation of typical Eastern North Pacific (ENP) gray whale migratory routes while the purple line demonstrates the probable northern Puget Sound (NPS) gray whale seasonal detour. (B) Plots illustrating the tidal stability of the study area from NOAA Station ID #9447659 in Everett, Washington. Top: variation in the mean daily feeding window (tidal height >4.5 feet, ∼1.37 m) since 1991 (see Materials and Methods and Table 1 for further description of terminology). Year is represented on the *x*-axis. The mean daily feeding window (MDFW) is represented on the *y*-axis as single points with the standard deviation shown as vertical error bars. Bottom: 2024 tidal height data (*y*-axis) on a daily 24 h scale. Blue lines represent days occurring during peak gray whale season in the NPS (1 March–31 May).

NPS gray whales have been increasing in number since the early 1990s, detouring nearly 250 km every year from traditional migratory paths into the northern Puget Sound (Fig. 2A) and staying an average of 3 months. Peak sightings occur between March and May, before individuals presumably resume their migration north (Calambokidis, 2017; Calambokidis et al., 2024), though some individuals have been reported in the area year-round. This region is characterized by large areas of planar or near-planar intertidal zones with fine sediment, subject to semidiurnal-mixed tidal fluctuations which vary in amplitude and duration more on shorter time scales (days, weeks) than on longer time scales (months, years) (Fig. 2B). Here, NPS gray whales forage on ghost shrimp (*Neotrypaea californiensis*), which densely inhabit these shallow and soft-bottomed intertidal regions, during sufficiently high tidal levels (Weitkamp et al., 1992; Pruitt and Donoghue, 2016). These feeding events leave large depressions (pits) on the intertidal that are accessible during suitably low tides and even observable in satellite imagery (Weitkamp et al., 1992; Calambokidis, 2017). The routine return of the same individuals and their predictable foraging behavior have provided a unique opportunity to perform mechanical studies on gray whale feeding behavior. We used whale-borne video and movement-sensing tags to quantify feeding rates and body kinematics during foraging as a function of the high-tide feeding window when abundant ghost shrimp are available in the seafloor. We hypothesized that gray whale kinematics reflect a suction-feeding strategy that maximizes the surface area of their unique skull morphology that interacts with the benthos. These results suggest that gray whale cranial morphology may facilitate benthic suction feeding in shallow coastal habitats, potentially opening new ecological niches in this lineage.

**Table 1.** Description of feeding-relevant terms and definitions used in this study

Term	Definition
Feeding roll	A departure from a neutral orientation (<10 deg in roll) where an individual maintained a lateral state (>90 deg in roll) for longer than 2 s before returning to a neutral position.
Feeding position	Sustained lateralization (>90 deg in roll) for longer than 2 s during which individual feeding events may occur.
Feeding event	Spikes in jerk in accelerometry data streams while the animal is in a feeding position that were assumed or camera validated to coincide with active feeding. Most likely the result of contact with the seafloor.
Foraging state	Periods of extended feeding within the same tidal cycle where individual feeding rolls are no more than 10 min apart and therefore include search and transit times.
Feeding window	Tidal heights above 4.5 feet (1.37 m), representing over 99% of the distribution of when feeding occurred in tag data streams and taken to generally represent the minimum tidal height required to initiate extended foraging.
FR/FWH	Feeding rate per hour of foraging window. Calculated by dividing the total number of feeding events for that deployment by the total hours of foraging window covered in that deployment.
Daily feeding rate	The estimated total number of feeding events per day. Calculated by multiplying the number of feeding events per foraging window hour (FR/FWH) by the total number of foraging window hours for that deployment day (not to be confused with the total number of hours covered by that deployment). This excludes deployments where the duration of feeding window coverage by that deployment was less than 2 h.
Minimum estimated benthic contact area (MEBCA)	The minimum estimated area (m^2^) of the whale skull model which came into contact with the contact plane (‘benthos’) during 3D model simulations of feeding rolls using tag data streams (depth, pitch, roll).

The ‘Term’ column represents the feeding-relevant terms used in the main body text while the ‘Definition’ column provides an expanded description of how that term was defined in this study.

## MATERIALS AND METHODS

### Cranial morphology

We compiled measurements of cranial morphology for baleen whale species from published datasets and from research done on specimens held by various museums, including standardized overhead images taken at the Los Angeles County Museum and the Smithsonian Institution. Physical measurements as described in Pyenson et al. (2013) include the bizygomatic width (BIZ), condylobasal length (CBL), curved length of the lower mandible (Cu) and the straight chord length of the lower mandible (Ch), which together characterize the overall aspect ratio of the skull and the relative curvature of the mandibles.

### Biologging data and feeding analysis

We deployed *n*=39 suction-cup attached Custom Animal Tracking Solutions (CATS) tags on 16 unique gray whales in the NPS in the years 2015–2016, 2021 and 2023–2024, resulting in approximately 19 days of multisensor data. See Table S2 for a full breakdown of deployment and feeding metadata. Earlier years (<2023) used pole-based methods for deployment (*n*=13) following methods in Goldbogen et al. (2006). A drone-based tag deployment (*n*=26) method was employed in more recent years (2023, 2024) following Wiley et al. (2023). All deployments were performed under National Marine Fisheries Service (NMFS) Permit No. 21678. Tag configuration varied but in general included accelerometry (200–800 Hz sampling rates), gyroscope (50 Hz) and magnetometer (50 Hz), with periodic video and acoustic recording as well as location-based sensing. Tags were outfitted with VHF transmitters and ARGOS satellite transmitters to be recovered via YAGI receivers once off the tagged animal. Data were downloaded via manufacturer (CATS)–user interfaces. Raw triaxial accelerometry data were downsampled to 10 Hz and processed following methods in Cade et al. (2021) for derivation of animal-frame pitch, roll, heading and norm jerk [absolute value of (acceleration/time)]. Jerk was smoothed to minimize noise for the following analyses (moving average, window size=5 s, sigma=1). We estimated speed using high-frequency accelerometer vibrations (tag jiggle) resulting from turbulence following Cade et al. (2017). All calculated speed values were relatively accurate within a deployment but were not well calibrated to absolute values as the *in situ* calibration method relies on periods of steep descents and ascents (>45 deg for prolonged periods) so that orientation-corrected depth rates could be compared with tag jiggle speed. For these shallow-diving animals, such calibration periods were absent. Location data was largely excluded from the reported analyses, as deployments differed in their GPS recording capabilities. However, the majority (*n*=27) of deployments used on-board GPS sensors and pseudo-track creation following Cade et al. (2021). Some (*n*=11) utilized customized CATS tags outfitted with Lotek SirTrack FASTGPS units, enhancing geolocation capacity. Geo-referenced pseudotracks were generated using a combination of boat-based focal follow positions, tag GPS data, animal heading, pitch, depth and estimated speed, and corrected for the land-based obstacles present in this near-shore, complex environment following Cade et al. (2021).

**Fig. 3.**
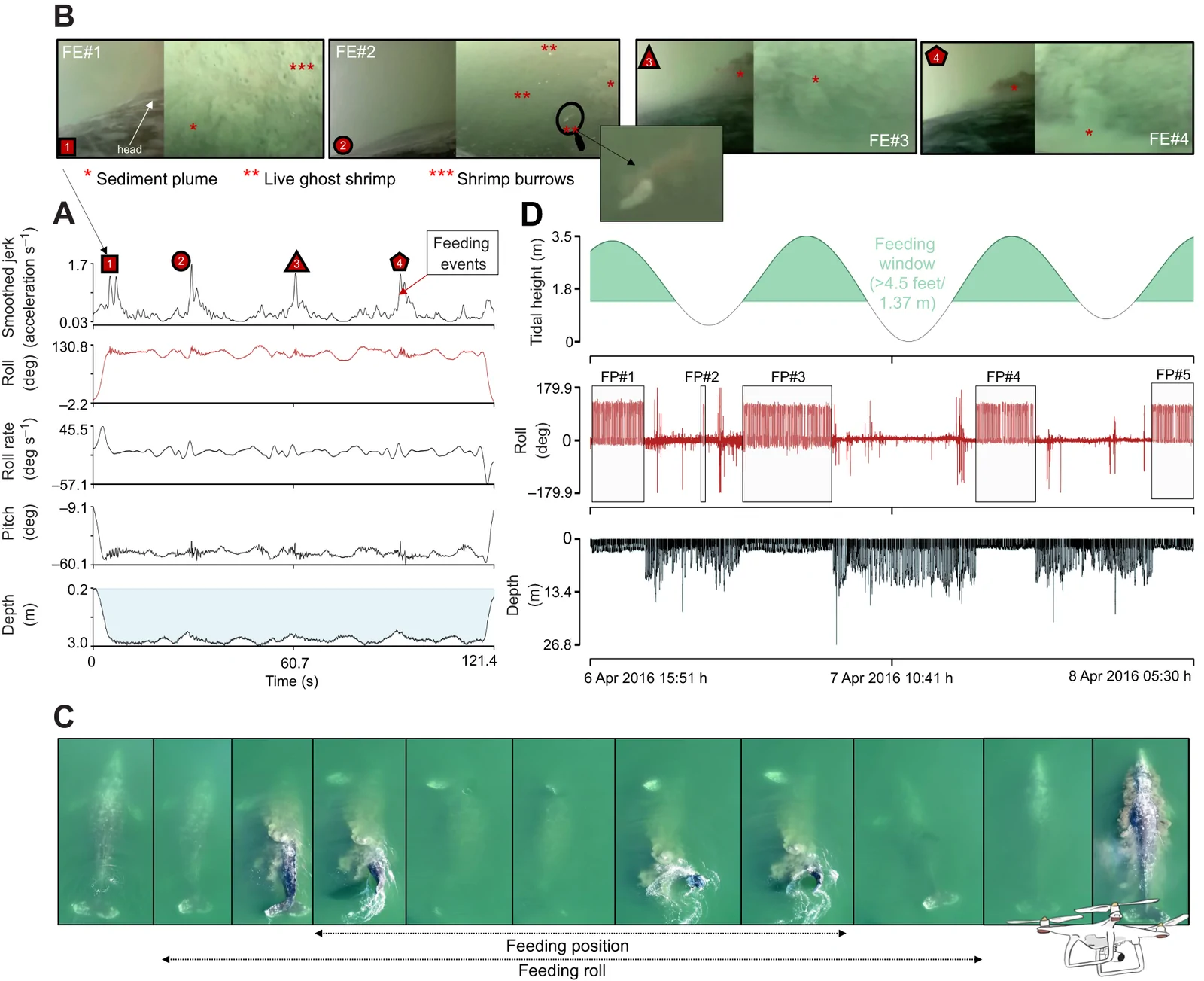
**Methods concerning NPS gray whale tag data foraging audit methods from a kinematic and observational perspective.** (A–C) A single feeding roll from CRC-21 from a kinematic (A), animal-borne tag camera recording (B) and drone (C) point of view. (A) Smoothed jerk (acceleration/time), roll, the rate of rolling (change in roll/time; with a low-pass 0.5 Hz Butterworth filter applied prior to calculation), pitch and depth of CRC-21. ‘Jerk’ signals associated with feeding events and their screenshots from respective video validation (B) are shown in the top subplot (smoothed jerk) as unique red symbols and numbers. Video screenshots of these events in B are further annotated with red asterisks noting where sediment plumes associated with seafloor contact (*), ghost shrimp (**) and their burrows (***) are observed. (C) Drone images with feeding position and feeding roll periods marked. (D) Whole deployment tag record from er20160406-21 demonstrating the tidal height, roll and depth as subplots. The feeding window is represented as the green shaded area in the top subplot 1 (tides). Foraging ‘periods’ (FP) or ‘states’ are annotated in the middle subplot (roll) with gray shaded boxes and their corresponding foraging period numbers.

Kinematic trends in depth, roll and the smoothed jerk (acceleration/time, window size 5 s) of tagged animals were analyzed to identify feeding rolls. Feeding rolls were defined as extended periods (>2 s) of lateralization (>90 deg in roll) (Fig. 3A,D). If an orientation of >90 deg was not maintained for more than 2 s, the individual was not considered to have entered a feeding position, and these rolls were excluded from the following analyses. Many deployments were also equipped with periods of video recording and where these coincided with potential feeding rolls (*n*=474), camera feeds were checked or completely audited (BORIS) for visual indicators of active feeding including observation of target prey, head movement, contact with the seafloor and/or creation of sediment plumes (Fig. 3B). Video validation of these feeding events (*n*=751) revealed (1) that a portion of individuals remained in an extended lateralized position during which multiple discrete feeding events occurred within a singular feeding roll (*n*=395), and (2) that active feeding events coincided with spikes in jerk in accelerometry data streams, likely due to contact with the seafloor (Fig. 3A,B; see also Movies 1 and 2). Thus, these spikes were used both to validate potential rolls as feeding events where cameras were not recording and to estimate feeding event rates for individuals (*n*=4) utilizing this extended roll strategy. This produced two estimates of foraging performance, (1) the number of feeding rolls, representing the number of times an individual initiated a roll to its side to feed, and (2) the number of feeding events, representing the number of spikes in jerk while the animal was in a feeding position. A linear regression was used to evaluate the relationship between the number of feeding events per feeding roll and the duration the whale spent in a feeding position, as a means of investigating the accuracy with which jerk signals may represent feeding events during periods with no available video recording.

Following audits of feeding rolls, broader-scale foraging states were defined as periods of extended feeding within the same tidal cycle, and durations were quantified as the time between the first and last feeding roll provided there was less than 10 min between individual feeding rolls (Fig. 3C), as this value was well above the average time between feeding rolls. Tidal characteristics were retrieved through publicly available tidal data (National Oceans and Atmospheric Administration, NOAA) using Everett Station ID #9447659 and scripts in Python to contextualize tidal dynamics of feeding observed via tag data. Over 99% (*n*=6168 out of 6225 total events) of feeding events occurred at tidal heights higher than 4.5 feet (∼1.37 m) (Fig. S1A), and so this value (>4.5 feet) was used to define a tidally based ‘feeding window’ during which active feeding was considered possible. Deployments that did not include at least 2 h of feeding window (*n*=11) were excluded from feeding rate calculations below to provide best estimates given variation in the tidal height at start of foraging. Feeding rates were first calculated by dividing the total number of feeding events by the total hours of feeding window covered by that deployment and thus represent the number of feeding events per hour of feeding window (FR/FWH). This allowed daily feeding rates to be estimated regardless of whether deployments spanned a 24 h period by extrapolating the FR/FWH to the total hours of feeding window for that deployment day. If deployments spanned over 24 h (*n*=3) and therefore had different daily feeding window durations, the mean daily feeding window duration was used to estimate daily feeding rates. The feeding rate per hour of foraging state (FR/FSH) was also provided by dividing the total number of feeding events for a given foraging state by that state's duration (hours). Finally, the inter-feed interval (IFI), or the time between feeding events, was calculated on a foraging state basis to avoid inflation due to significant time between foraging (tidal) windows.

### 3D modeling and minimum estimated benthic contact area

**Fig. 4.**
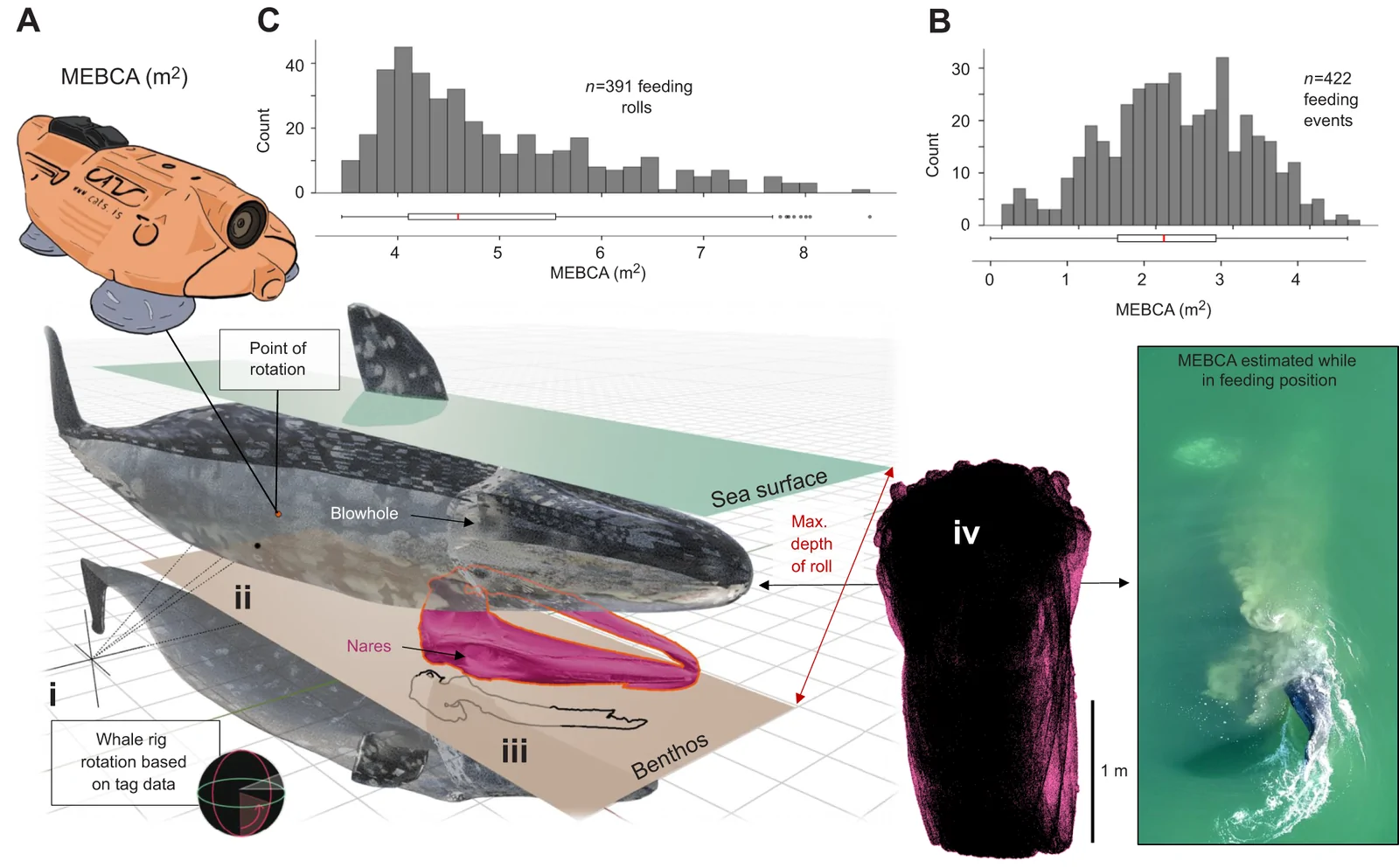
**Methods and results of simulation-based estimation of the minimum estimated benthic contact area (MEBCA).** (A) An exploded view of the whale body and skull model during a single frame of the simulation. The skull model is set within the whole-body model, and both are parented to a rig, a non-object point of gravity used to drive both the skull and whole-body models (i). The contact plane (ii) is set at the maximum depth of the feeding roll, and the rig is driven using pitch, roll and heading tag data. The contact plane bisects the skull model on a by-frame basis, and pixels occurring below the plane (iii) are cast onto it. At the end of the simulation, the cast imprint is patched, yielding a morphologically corrected imprint of the total area falling below the plane (iv) where black pixels represent the raw contact pixels and pink pixels represent the patched areas. (B) A histogram and boxplot (showing median, upper and lower quartiles and 1.5× the interquartile range) of the resulting mean uncorrected MEBCA in the 2 s period around feeding events. (C) A histogram and boxplot of the MEBCA for the entire feeding roll.

A 3D model of a gray whale skull was obtained from SketchFab (courtesy of Vic DeLeon). This model was built from an adult specimen at the San Diego Natural History Museum using the phone-based Polycam app. It was then parented to a manually built gray whale model to allow for multi-axis rotation as documented in tag data. The point of rotation was set on the midline dorsal aspect of the whale model, simulating typical tag placement, and both the skull and whole-body model were parented to a rig to allow for simultaneous rotation (Fig. 4Ai). Custom-written scripts in Python were executed in Blender 4.3.5 to animate the skull model with tag data for a randomly selected 10% subset of feeding rolls (*n*=391) and the feeding events occurring therein (*n*=422). The environment was scaled to the data's 1 m spatial dimension (1 Blender Unit=1 m). For each feeding roll, the whale model was translated according to downsampled (1 Hz) pitch, roll, heading and depth data. These data were smoothed using a Gaussian kernel smoother (sigma=2). Vertices were projected orthogonally onto a horizontal ‘contact plane’ positioned at the maximum depth of the feeding roll, simulating the sea floor (Fig. 4Aii). This was rasterized at a resolution of 1024×*n* pixels with a 5% relative padding, to account for variation in spatial scale between events, yielding an effective pixel size between 1 and 2 mm^2^. A binary mask was generated marking all pixels contacted by any vertex in the skull model, not the whale model, given the higher resolution of the skull model. For every second frame, each mesh vertex was transformed into *x*–*y* positions and cast onto a binary 2D grid (‘contact plane’) (Fig. 4Aiii). A single-pass morphological dilation (3×3) followed by hole-fill was applied to the binary mask to account for small gaps between vertices and holes on the skull mesh, yielding a morphologically corrected mask (Fig. 4Aiv). Contact areas were then computed as the sum of the pixel area for each mask with both raw and corrected minimum estimated benthic contact area (m^2^) (MEBCA) reported, along with the percentage difference between the two (Eqn 1):

(1) $$A_{\mathrm{raw}},A_{\mathrm{corr}}=\sum_{i}\mathrm{mas}k_{i}\mathrm{\Delta x}\mathrm{\Delta y},$$

where *A*_raw_ represents the sum area of pixels before correction and *A*_corr_ is the sum area after morphological correction is applied; mask*_i_* represents the binary mask dimension at every *i*-th feeding roll while Δ*x* and Δ*y* represent the change in *x*–*y* dimension between translated frames in vertices in the skull model.

Diagnostic images of the mask with a 1 m scale bar were then exported for visual inspection. Several diagnostic checks were performed to assess produced calculations, precision and accuracy. Geometric accuracy was assessed by replacing the skull model with simple shapes (flat plane, circle, rectangle) and inspecting the computed rasterized area with expected results. The effect of contact plane resolution was inspected by running simulations at resolutions between 256 and 2048 pixels and visually inspecting plots of resolution relative to estimated area. Temporal consistency across frames was assessed by recomputing the instantaneous area per frame instead of the union, and plotting the mean, minimum and maximum to visually inspect for smoothness.

## RESULTS

Tag deployments ranged from less than 1 h to approximately 36 h, with a mean±s.d. length of 11.4±9.78 h. Most individuals (*n*=12 out of 16) were tagged on multiple occasions, with an average of 2.8 deployments per individual (maximum 5 on individual CRC-383). Of these, 25 deployments recorded foraging behavior, resulting in a total of 420.96 h of data from foraging individuals. Tags recorded 128.59 h of NPS gray whales in a foraging state (*n*=86 individual states), with a mean number of foraging states per individual of 3, lasting an average of 1.47 h in duration. Many (*n*=19 out of 86) foraging states were cut short by tag displacement while feeding. All deployments coincided with semidiurnal mixed tidal cycle types, characteristic of this region (see Fig. 2B), with heights ranging from 0.4 to 3.5 m.

### Spatial and temporal characteristics

The feeding audit resulted in a total of 6317 feeding events occurring in 4002 discrete rolling events. Feeding rates per hour of feeding window averaged 21±12.3 h^−1^ (mean±s.d.) while feeding rates per hour of foraging state were 43.9±15.6 h^−1^, with only deployments spanning at least 2 h of the feeding window (e.g. tidal height >4.5 feet, ∼1.37 m) included in all feeding rate estimates. Extrapolating these hourly values to the available feeding window hours for each deployment (Table S2, mean daily feeding window duration) provided an estimated mean (±s.d.) daily feeding rate of 292.2±153.5 feeding events per day. The mean IFI within foraging states was 179.7±78.8 s (range of 595 s). Individuals used on average 38±28% of the available daily feeding window, with an average of 47.3% when weighted by deployment length to account for potential bias as a result of short deployments.

The MEBCA in the 2 s period around feeding events was 1.9±1.2 m^2^ (Fig. 4B) while the MEBCA per feeding roll was 4.9±1.1 m^2^ (Fig. 4C). Raster precision across different contact plane resolutions was <1%, converging at a resolution of over 1024 pixels. Finally, the mean daily area foraged per individual was estimated at 1332.6±817.6 m^2^ by multiplying the mean daily feeding rate and the mean MEBCA per feeding roll.

### Biomechanics

**Fig. 5.**
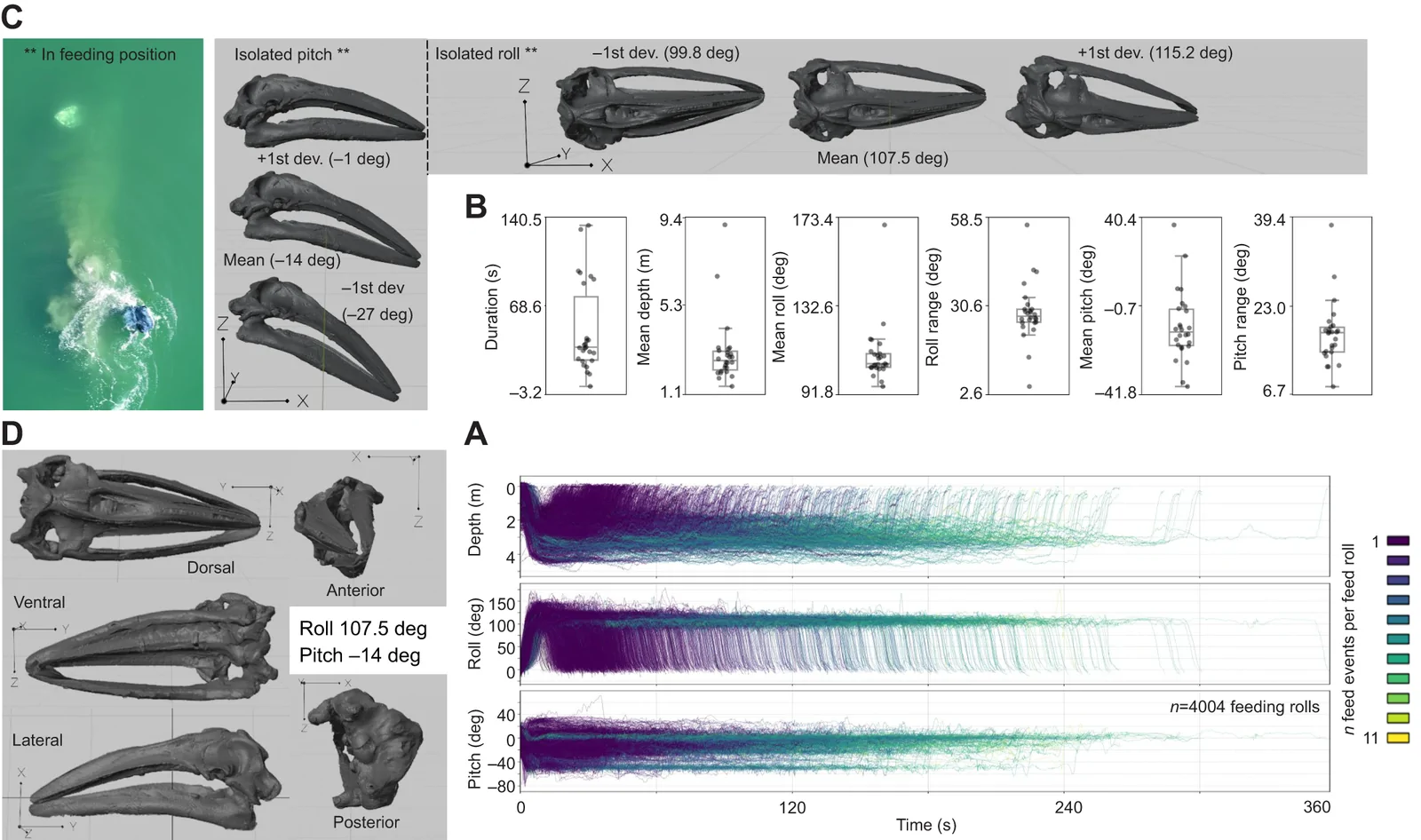
**Feeding mechanics from tag-based data.** (A) Kinematics of feeding NPS gray whales colored by the number of feeding events for that feeding roll, where subplots demonstrate depth (top), roll (middle) and pitch (bottom) of all feeding rolls relative to the feeding roll duration. (B) Boxplots (median, upper and lower quartiles and 1.5× the interquartile range) summarizing the distribution of key mechanics while in a feeding position. From left to right, subplots represent the duration spent in a feeding position, the mean depth, the range in depth, the mean pitch and the range in pitch while an animal is in a feeding position. (C) The whale skull model rotated to mean±1 s.d. isolated pitch and roll orientations. (D) Multiple views (dorsal, ventral, lateral, posterior and anterior) of the skull model in average pitch and roll orientation in a feeding position.

The body orientations of whales (Fig. 5) while feeding occurred at a mean (±s.d.) depth of 2.5±0.74 m (range 16.3 m), exclusively right-sided (circular mean 107.5±7.8 deg, range 96.1 deg) and with a mean duration of 43.3±45.6 s (range 341.4 s). Individuals were mostly negatively pitched (circular mean −14±13 deg, range 98.1 deg) while in a feeding position. Mean circular heading of feeding rolls was −5.1±76.6 deg, with a circular range of 194.9 deg. Mean circular heading in a foraging state was 16.28±62.7 deg with a range of 238.1 deg. The mean standard deviation in heading during foraging states was 63.5 deg (range of 104 deg). The number of feeding events while in a feeding position ranged from 1 to 11 (mean±s.d. 1.5±1.3) (Fig. 5A). The relationship between the number of feeding events performed during a feeding roll and the duration that individual spent in a feeding position was strongly linear (*r*^2^=0.75) (Fig. S1B).

### Environmental dynamics

Feeding occurred at a mean (±s.d.) tidal height of 9.3±1.85 feet (∼2.83±0.56 m) with a range of 10.4 feet (∼3.17 m) (Fig. S1A). Foraging was initiated at an average tidal height of 7.4 feet (∼2.26 m) on predominantly incoming tides (*n*=38) compared with outgoing tides (*n*=2). Feeding window duration [e.g. tidal heights >4.5 feet (1.37 m)] during days where tag deployments occurred ranged from 10.85 to 16.87 h a day with an average of 13.84±2.01 h.

### Cranial morphology

Cranial measurements were performed on the skeletal remains of 55 specimens from nearly all species (*n*=10) of mysticetes held by several institutions. A large number of the specimens were bowhead (*n*=10) and gray (*n*=14) whales. Mean (±s.d.) gray whale CBL/BIZ was 2.19±0.16 while Cu/Ch, a measure of mandible curvature, was 1.01±0.008. Both skim- and lunge-feeding species had a mean CBL/BIZ of 1.92±0.2, while lunge feeders had a Cu/Ch of 1.04±0.02 and skim feeders had a mean Cu/Ch of 1.06±0.02. See Table S1 for a complete list of measurements by species.

## DISCUSSION

### Characteristics of benthic foraging on ghost shrimp

Our study provides an integrative method of estimating the spatial characteristics of benthic foraging using animal-borne data and first measures of feeding rates for gray whales. These lay the foundation for future studies concerning foraging energetics while providing ecological context for the evolution of large body size. We show, by integrating spatial and temporal measures of foraging effort, that an individual gray whale may forage on the seafloor over an average area of 1332.6±817.6 m^2^ per day (mean±s.d.). Our estimates of seafloor area per feeding roll (4.9±1.1 m^2^) were within reported measurements of gray whale feeding dimensions both within the study area and in other areas. Weitkamp et al. (1992) physically measured feeding pits of an unreported sample size in the NPS, providing mean individual pit sizes of 3.4±0.3 and 3.7±1.3 m^2^ in 1990 and 1991, respectively. Nerini (1984) reported mean areas of under 5.3 m^2^ for what they considered ‘fresh’ pits in the Bering Sea using side-scan sonar. Determining the age of features in the benthos is logistically challenging and, as such, both studies relied on anecdotal evidence, such as clear edging, to discern fresh feeding events despite the added measurement uncertainty relevant to feature age. Our method transcends the need for aging by producing estimates from real-time events but lacks the assurance of measuring physical events in real space. The similarity between spatial measurements of foraging behavior despite the different estimate methods (tag-based, physical, side-scan sonar) is promising and may indicate that despite the numerous considerations, tag-based and model-integrated measurements may be a reliable estimate of spatial dimensions of foraging in this species. Quantifying area foraged in the benthos is important because this can be integrated with prey density measurements to estimate prey intake, enabling foraging energetics to be understood in broader ecological contexts.

These results provide a promising avenue to quantifying the spatial dimensions of foraging using tag data, but we note that our approach is likely to reflect minimum bounds in space for any given feeding event. The most obvious source of uncertainty comes from our use of a skull model, instead of a whole-body model, to calculate the minimum estimated benthic contact area. We opted to use this skull model given the lower quality and lack of transparent sourcing of comparable whole-body models. Further, our model simulations do not allow independent flexion and rotation and therefore restrict the potential range of motion along several points on the whale model, many of which are important to the mechanical processes of whale feeding. First, cervical vertebrae are unfused in both gray whales and rorquals, allowing for a higher range of motion than possible by fixing the skull model within a whole-body model. Second, gape angle has been well studied using tag and drone technologies for some lunge-feeding rorqual whales such as the blue whale (Torres et al., 2020) and fin whale (Goldbogen et al., 2010) and is an important component of the mechanical processes involved in efficient lunge feeding. Gray whale cranial morphology and mandible musculature, in contrast, suggest a limited gape angle compared with that of rorquals (El Adli and Deméré, 2015), and empirical reports suggest a near-closed gape angle while foraging (Ray and Schevill, 1974). As such, we opted for a completely closed mouth during simulations of contact area. Even with the mouth closed, the mandibles could be rotated about their long axis to modulate suction forces that impact the contact area. Paired drone-based monitoring of tagged animal foraging and physical measurements of the pits they create could enable a better understanding of the impact of skeletal models and gape on spatial measurements. Further, our simulations used a model based on a single adult male specimen, while our tag dataset was composed of both males and females of varying lengths. Potential differences in foraging behavior relative to sex and morphology are poorly understood for baleen whale species, but are common in both terrestrial (passerine birds: Holmes, 1986; large herbivores: du Toit, 2005) and marine species (e.g. Adelie penguins: Clarke et al., 1998; grey seals: Beck et al., 2007; northern elephant seals: Le Boeuf et al., 1993). Future work could include drone-based measurements of total length to scale the skull to an individual's morphology, which may reveal differences in foraging performance. Finally, our simulation assumes a completely flat plane, but benthic environments may not be perfectly flat. Nevertheless, ghost shrimp habitat in the study area is extremely flat (Fig. 2Ai), making it an ideal environment for these kinds of estimations. Advances in biologging technology, specifically in GPS capabilities, could allow for the integration of high-resolution digital elevation models (like the one in Fig. 2Ai) with simulations, to account for non-planar locations and variable prey environments.

### Foraging performance and feeding rates

Characterizing foraging behavior using non-invasive techniques is critical to understanding how organisms function and interact with their environment. We demonstrate that accelerometer-derived ‘jerk’ (or the acceleration rate of change) signals co-occur with individual feeding events in NPS gray whales, using concurrent whale-borne video as validation. We observed multiple feeding events while whales were engaged in a single rolling maneuver, and each jerk signal, with several occurring during a single roll, appears to reflect contact with the seafloor as the mouth engulfed sediment and prey. Given all annotated spikes in jerk coincided with evidence of feeding events in the video footage (sediment plume, displaced prey), we suspect the likelihood of false positives to be low in datasets where there were no video recordings. The magnitude of an accelerometer-derived jerk signal, however, is strongly dependent on tag placement and other factors such as sampling rate (Ydesen et al., 2014). We found that the relationship between the duration a whale spent in a feeding position (rolled >90 deg for >2 s) and the number of feeding events occurring therein was strongly linear (*r*^2^=0.75), with minimal outliers (see Fig. S1B). This relationship may reflect a consistent ‘handling time’, or a biomechanical constraint related to the filtering of water and sediment from the mouth.

Our results show that despite being tidally restricted, gray whales foraging in the NPS were not in a foraging state throughout the entire tidally available window. Instead, gray whales were in a foraging state on average for less than half (47.3%) of the available feeding window hours. This may suggest most individuals are either not motivated to utilize the entire feeding window or are experiencing other physiological and/or environmental limits not evaluated in this study. The tidal characteristics at the point of foraging vary both spatially and temporally. For instance, comparing deployments of similar lengths but with differing feeding window usage indicates that low tidal characteristics (low tide level and the amount of time at lower tides) varies (Fig. S2). Individuals may alter their foraging effort in response to the tidal environment due to changes in daily low and high tidal height durations, which are more dramatic on the scale of weeks than days.

Further, our characterization of foraging state includes behaviors indirectly associated with active feeding, such as short duration (<10 min) searching, dive recovery, repositioning and/or food handling, all of which reduce the time available to actively feed. This unexploited foraging opportunity may suggest that gray whales are not limited by the prey environment (i.e. access or patch depletion) as experienced by lunge-feeding rorquals during diel vertical migration of krill and/or patch depletion (Oleson et al., 2007; Goldbogen et al., 2015a). However, foraging effort may be influenced by prey dynamics on larger temporal–spatial scales (weeks to months), given the ecology of ghost shrimp.

NPS gray whales suction feed in shallow waters (mean 2.5 m), leading us to suspect they experience reduced vertical transit and pressure-related costs relative to deeper foraging. The spatial and temporal separation of critical resources (food, air) for most marine mammals has led others to conclude that individuals should exhibit optimal foraging behavior (Benoit-Bird, 2023). Foraging at such shallow depths may enable NPS gray whales to maximize intake of prey only accessible during sufficiently high tidal levels while minimizing locomotive costs, which could explain why gray whales in this study used at most half of the available feeding window. Still, anecdotal evidence suggests ghost shrimp are more mobile and capable of evasion (see Fig. 3B and Movies 1 and 2) when compared with typical gray whale prey throughout the rest of their range (amphipods, mysid shrimp). Individuals may not be particularly adept at capturing their intended prey, resulting in decreased energy consumption despite low investment. If true, this variation in capture efficiency may underlie the highly variable foraging rates we recorded in tag data. Despite this variation, our measured feeding rates extrapolated to the daily scale (mean 292 feeding events per day) were similar to those reported for other large baleen whale species, such as blue whales and fin whales foraging on krill in the eastern North Pacific (estimated 178–270 and 159–302 foraging lunges per day, respectively) (Savoca et al., 2021) and minke whale whales (Cade et al., 2023). While this similarity in count-based foraging effort amongst these species is interesting, given their drastically different foraging ecologies, these same differences in feeding mechanics and physiology warrant more expansive studies concerning the energetic dynamics (prey intake, energy expenditure) of NPS whale foraging that may provide insight into their adaptive and evolutionary history in comparison to rorqual whales.

NPS gray whales visit the study area seasonally, using it as a stop-over foraging ground before presumably continuing their migration north, and thus the individual's body condition may influence the duration spent foraging. Condition-dependent variation in foraging effort may arise as a result of differences in buoyancy, which can increase or decrease the energetic cost of shallow water foraging (Rosen et al., 2007; Bird et al., 2024b, 2025) or, more simply, because whales with better body condition may have less incentive to carry out extended foraging than if they have poorer body condition. Future work integrating estimates of body condition and foraging energetics with tag-based foraging characteristics could help elucidate potential limits.

### Tag-based kinematics

Intertidal foraging by gray whales in the NPS is characterized by extended side-rolls with a slightly negative pitch followed by a return to a horizontal position (pitch ∼0 deg) as the rolling event is terminated (Fig. 5). These kinematic signatures while foraging (lateralization with slightly negative pitch) likely reflect how gray whales use straight, paddle-like lower mandibles and an elongated, high aspect ratio skull (Fig. 1) to maximize surface area contact with the seafloor. With contact in this orientation (Fig. 4), gray whales can then depress the hyolingual musculature to create negative pressure and direct the flow of water–sediment–prey into their mouths (Werth, 2007; Kienle et al., 2015). Paired with few, large ventral throat grooves, gray whales appear specialized for manipulating the benthos with pulsatile suction feeding. This strategy contrasts with the ecomorphology of rorquals, consisting of an extensive ventral groove system that accommodates extreme engulfment capacity (Goldbogen et al., 2019; Shadwick et al., 2019; Kahane-Rapport et al., 2020). These form–function trade-offs beg questions concerning how the efficiency of foraging on different prey in vastly different habitats compares both between gray whale sub-groups with different foraging ecologies (e.g. Pacific Coast Feeding Group and NPS gray whales) and with lunge-feeding whale species. Both groups (gray whales, rorquals) appear to have specialized filter-feeding morphologies while also being prey generalists, with some exceptions such as blue whales only feeding on krill. Integrating multiple high-resolution species models in comparative biomechanical simulations could help elucidate the energetic underpinnings of this form–function relationship driving speciation and niche partitioning.

Many cetacean species have been documented using a wide range of rolling maneuvers during foraging, indicating its broad utility in prey capture. Humpback whales (*Megaptera novaeangliae*) in the Southern Gulf of Maine use side rolls of 80–120 deg to feed along the seafloor on sand lance (Hain et al., 1995; Ware et al., 2014) in tens of meters of water depth. Common bottlenose dolphins (*Tursiops truncatus*) use coordinated side rolls to capture schooling fish at low tide in coastal salt marshes of North Carolina (Kleinclaus, 2022). In more offshore habitats, multiple rorqual species (Balaenopteridae) execute rolls while lunge feeding on krill at or near the sea surface (Kot et al., 2014; Owen et al., 2016; Torres et al., 2020; Izadi et al., 2022; Rychwalski and Herr, 2024) as well as in deeper parts of the epipelagic (Goldbogen et al., 2006; Cade et al., 2016). The degree of roll during a lunge varies extensively within and among rorqual species, from minimal (<45 deg) to lateral (∼90 deg), inverted (∼180 deg) and up to full 360 deg barrel rolls (Simon et al., 2012; Goldbogen et al., 2013; Friedlaender et al., 2017). Much of this variation remains unexplained, though in blue whales more acrobatic rolling was observed when whales were foraging on shallower, lower density krill patches (Goldbogen et al., 2015a,b). Whale-borne video suggests that these types of rolls may enhance visual survey of the prey field from beneath (Goldbogen et al., 2013). In the deep sea, echolocating sperm whales and beaked whales appear to roll to adopt specific approach angles for echo-guided prey capture (Fais et al., 2016; Madsen et al., 2013), which in some cases occurs along the seafloor (Arranz et al., 2011, 2019). In total, these studies show that rolling is a common feature in cetacean foraging as they capture throughout the water column, from gray whales in the intertidal to beaked whales on the abyssal seafloor (Marsh et al., 2018).

In sum, our study furthers our understanding of gray whale foraging ecology, physiology and morphology by using biologging data and 3D modeling techniques to characterize foraging behavior in this unique group of whales foraging in a tidally constrained habitat. Although ghost shrimp are a novel prey item for gray whales, at least in recorded history, they are similar in ecology to the tube-dwelling amphipods foraged upon by gray whales in the higher latitudes, presumably the prey type gray whales evolved to exploit (Pyenson and Lindberg, 2011). Both organisms occur in extremely high densities in the fine benthic sediments of either intertidal (shrimp) zones (Weitkamp et al., 1992; Pruitt and Donoghue, 2016) or large basins/continental shelves (amphipods) (Stewart et al., 2023; Moore et al., 2022; Highsmith and Coyle, 1992). Finer scale comparisons of the energetic value (energy density) and abundance (individuals per area of the seafloor) of these target species are needed to better understand the physiological drivers and environmental limits in this predator–prey system.

## Supplementary Material

10.1242/jexbio.252034_sup1 Supplementary information
