# Supplementary material for: Kinematics of gray whale (Eschrichtius robustus) foraging on infaunal ghost shrimp (Neotrypaea californiensis)
Source: J Exp Biol. 2026 Sep 18;229(18):jeb252034. doi: 10.1242/jeb.252034 (PMC13615682; doi:10.1242/jeb.252034)
Supplement: Supplementary file 1 [file jexbio-229-252034-s1.pdf]

Table S1. Comparative cranial and mandibular skeletal measurements of baleen whales grouped by extant species (“Species”, “Common Name”). “N”, the number of measured specimens. “CBL/BIZ”, mean ratio of Condylobasal Length (CBL) (skull length) to Bizygomatic Width (skull width) (BIZ). “Cu/Ch”, the mean ratio of lower mandible curved length (Cu) to its chord/straight length (Ch). “Primary Feeding Mode”, the primary foraging strategy utilized by the species as coded in Figure 1 of the manuscript.

Species	Common Name	N	CBL/BIZ	Cu/Ch	Primary Feeding Mode
Balaenoptera musculus	Blue whale	4	1.92	1.06	Lunge
Balaenoptera edeni	Bryde's whale	5	2.04	1.04	Lunge
Balaenoptera physalus	Fin whale	3	2.16	1.04	Lunge
Balaenoptera borealis	Sei whale	2	2.25	1.02	Lunge
Meganoptera novaeangliae	Humpback whale	3	1.6	1.07	Lunge
Balaenoptera acutorostrata	Common minke whale	8	1.8	1.04	Lunge
Balaena mysticetus	Bowhead whale	10	2.01	1.07	Skim
Caperea marginata	Pygmy right whale	2	1.88	1.02	Skim
Eubalaena glacialis	North Atlantic right whale	5	1.75	1.07	Skim
Eschrichtius robustus	Gray whale	14	2.19	1.01	Benthic

Journal of Experimental Biology • Supplementary information

Table S2. NPS gray whale tag deployment metadata. "Deployment ID", shorthand notation identifying the tag deployment. Includes the shorthand species scientific name "er", the year, date, month, followed by the tag number. "CRC-ID", the tagged individual's Cascadia Research Collective identification number. "# Deployments This Whale", the number of times this animal has been tagged. "Tag On DateTime", the date-time the tag was deployed. "Tag Off DateTime", the date-time the tag fell off or stopped recording. "Deployment Duration (Hours)" the length of this tag deployment in hours. "Feeding Recorded?", whether the tagged individual was found to be in a foraging state during this deployment. "*n* Foraging States", the total number of foraging states or periods. "*n* Feeding Rolls", number of feeding rolls this deployment. "*n* Feeding Events", number of feeding events in this deployment. "Total Foraging State Duration (hours)", the total hours this whale was observed in a foraging state for this deployment. "Feeding Rate per Hour of FS", the total number of feeding events divided by the total number of hours this whale was in a foraging state. "Feeding Rate per Hour of Feeding Window", the total number of feeding events divided by the total number of feeding window hours covered by this deployment. "Mean Daily Feeding Window Duration (Hr)", the mean (if multiple days are covered) duration in hours of the feeding window of this deployment day. "Estimated Daily Feeding Rate", the extrapolated daily feeding rate based on the feeding rate per hour of feeding window and the mean feeding window duration for the day. "% FW Used", the proportion (as percent) of the feeding window covered by the deployment that the tagged individual was found in a foraging state.

Deployment ID	CRC-ID #	# Deployments This Whale	Tag On	Tag Off/Data End	Deployment Duration (Hours)	Feeding Recorded ?	*n* Foraging States	*n* Feeding Rolls	*n* Feeding Events	Total Foraging State Duration (hours)	Feeding Rate per Hour of FS	Feeding Rate per Hour of FW	Mean Daily Feeding Window Duration (Hr)	Estimated Daily Feeding Rate	% FW Used
er150417-3	22	3	04/17/15 08:52:59	04/18/15 03:40:04	17.4	Y	3	257	330	8.6	38.4	30.6	11.27	344.4	80%
er150419-3	22	3	4/19/15 08:55:23	4/19/15 08:56:04	0.01	N	0	0	0	0	N/A	N/A	11.93	N/A	0%
er150419-5	383	5	4/19/15 13:35:52	04/19/15 15:59:00	2.4	N	0	0	0	0	N/A	N/A	11.93	N/A	0%
er160325-22	723	3	03/25/16 07:48:12	03/26/16 07:34:14	23.1	Y	2	174	558	9.1	61.3	35.3	14.69	518.8	58%
er160325-25a	49	2	03/25/16 14:38:50	3/25/16 15:36:56	1.0	N	0	0	0	0	N/A	N/A	14.32	N/A	0%
er160325-25b	383	5	03/25/16 18:01:41	03/26/16 07:02:12	13.0	Y	3	210	285	5.1	55.9	27.1	14.69	398.7	49%
er160406-21	723	3	04/06/16 15:51:55	04/08/16 05:30:14	37.6	Y	5	263	911	15.0	60.7	36.9	12.07	445.2	61%
er160406-22	21	2	04/06/16 14:28:50	04/07/16 08:39:23	18.2	Y	5	387	450	7.8	58.1	35.7	11.12	397.1	62%
er160407-25	383	5	04/07/16 13:55:34	04/07/16 18:21:52	4.4	Y	1	56	63	1.2	52.1	16.2	10.85	175.3	31%
er160505-22	723	3	05/05/16 13:17:44	05/05/16 17:41:46	4.4	Y	1	24	106	2.0	53.0	26.5	11.98	317.5	50%
er160505-25	49	2	05/05/16 13:53:01	05/05/16 20:21:26	6.5	Y	1	83	91	2.8	32.7	14.0	11.98	167.7	43%
er210415-58	531	2	04/15/21 17:45:20	04/16/21 21:43:14	28.0	Y	10	244	265	7.1	37.3	24.5	15.98	392.1	66%
er230414-55	53	2	04/14/23 14:21:57	04/14/23 15:55:11	1.6	N	0	0	0	0.0	N/A	N/A	16.48	N/A	0%
er230414-70	21	2	04/14/23 13:42:33	04/14/23 20:29:02	6.8	N	0	0	0	0.0	N/A	N/A	16.48	N/A	0%
er230415-55	2249	3	04/15/23 11:12:00	04/15/23 14:53:58	3.7	Y	2	5	5	0.6	8.8	1.4	16.87	22.8	15%
er230415-71	2362	3	04/15/23 10:31:49	04/16/23 14:26:31	27.9	Y	1	179	195	5.1	38.5	9.0	16.68	149.9	23%
er230415-88	383	5	04/15/23 10:47:24	4/15/23 14:33:23	3.8	Y	2	1	1	0.0	0.0	0.3	16.87	64.1	0%
er230416-55	543	2	04/16/23 09:29:49	04/17/23 01:00:35	15.5	Y	9	141	154	5.2	29.7	15.2	15.99	243.8	51%
er230416-70	531	2	04/16/23 09:53:20	04/17/23 00:27:46	14.6	Y	1	206	206	6.6	31.4	22.6	15.99	362.0	72%
er230420-55	2362	3	4/20/23 10:47:31	04/20/23 19:19:57	8.5	N	0	0	0	0.0	N/A	0.0	13.63	0.0	0%
er230421-88	356	1	04/21/23 13:06:34	04/22/23 00:53:15	11.8	Y	4	139	144	4.07	35.4	16.0	16.06	257.0	45%
er240407-55	543	2	04/07/24 10:13:34	04/07/24 10:54:58	0.7	N	0	0	0	0	N/A	N/A	10.97	N/A	N/A
er240407-70	2356	3	04/07/24 08:50:42	04/07/24 14:23:22	5.5	Y	1	19	19	0.45	42.2	15.8	10.97	173.7	38%
er240407-71	56	4	04/07/24 12:53:49	04/08/24 10:54:38	22.0	Y	2	129	407	7.17	56.8	26.1	10.98	286.5	46%
er240409-48	383	5	04/09/24 14:54:45	04/09/24 20:17:37	5.4	Y	1	148	173	3	57.7	34.6	12.16	420.7	60%
er240409-49	22	3	04/09/24 11:46:16	04/09/24 15:58:47	4.2	N	0	0	0	0	N/A	0.0	12.16	N/A	0%
er240409-70	2259	2	04/09/24 09:56:01	04/09/24 14:51:48	4.9	N	0	0	0	0	N/A	N/A	12.16	N/A	N/A
er240409-71	2356	3	04/09/24 10:45:56	04/10/24 04:03:04	17.3	Y	1	239	271	5.32	50.9	23.0	12.52	287.5	45%
er240409-T4	56	4	04/09/24 13:56:01	04/09/24 19:07:43	5.2	Y	2	39	109	2.31	47.2	27.9	12.16	339.9	59%
er240410-49	2249	3	04/10/24 11:50:27	04/11/24 19:32:13	31.7	Y	7	496	546	8.59	63.6	26.5	14.41	381.9	42%
er240410-70a	2249	3	04/10/24 10:07:10	04/10/24 10:31:55	0.4	N	0	0	0	N/A	N/A	N/A	12.87	N/A	N/A
er240410-70b	56	4	04/10/24 11:04:29	04/11/24 07:15:03	20.2	Y	4	155	395	8.4	47.0	26.2	14.41	377.0	56%
er240410-T4	2356	3	04/10/24 11:17:10	04/10/24 19:45:54	8.5	Y	3	118	133	3.48	38.2	36.9	12.87	475.5	97%
er240507-70	2259	2	data issue, not analyzed												
er240507-48	2441	1	05/07/24 10:54:28	05/08/24 03:18:35	16.4	Y	2	42	42	1.19	35.3	3.3	15.11	49.6	9%
er240507-71	56	4	05/07/24 10:46:30	05/08/24 09:10:52	22.4	Y	5	83	291	5.66	51.4	16.3	15.11	245.6	32%
er240508-49	2362	3	05/08/24 08:35:45	05/08/24 19:34:18	11.0	Y	2	165	167	2.92	57.2	38.8	15.30	594.2	68%
er240508-T4	53	2	05/08/24 10:44:22	05/08/24 11:49:40	1.1	N	0	0	0	0	N/A	N/A	15.30	N/A	N/A
er240509-71	2557	1	05/09/24 10:06:51	05/09/24 15:49:08	5.7	N	0	0	0	0	N/A	N/A	15.00	N/A	N/A

Journal of Experimental Biology • Supplementary information

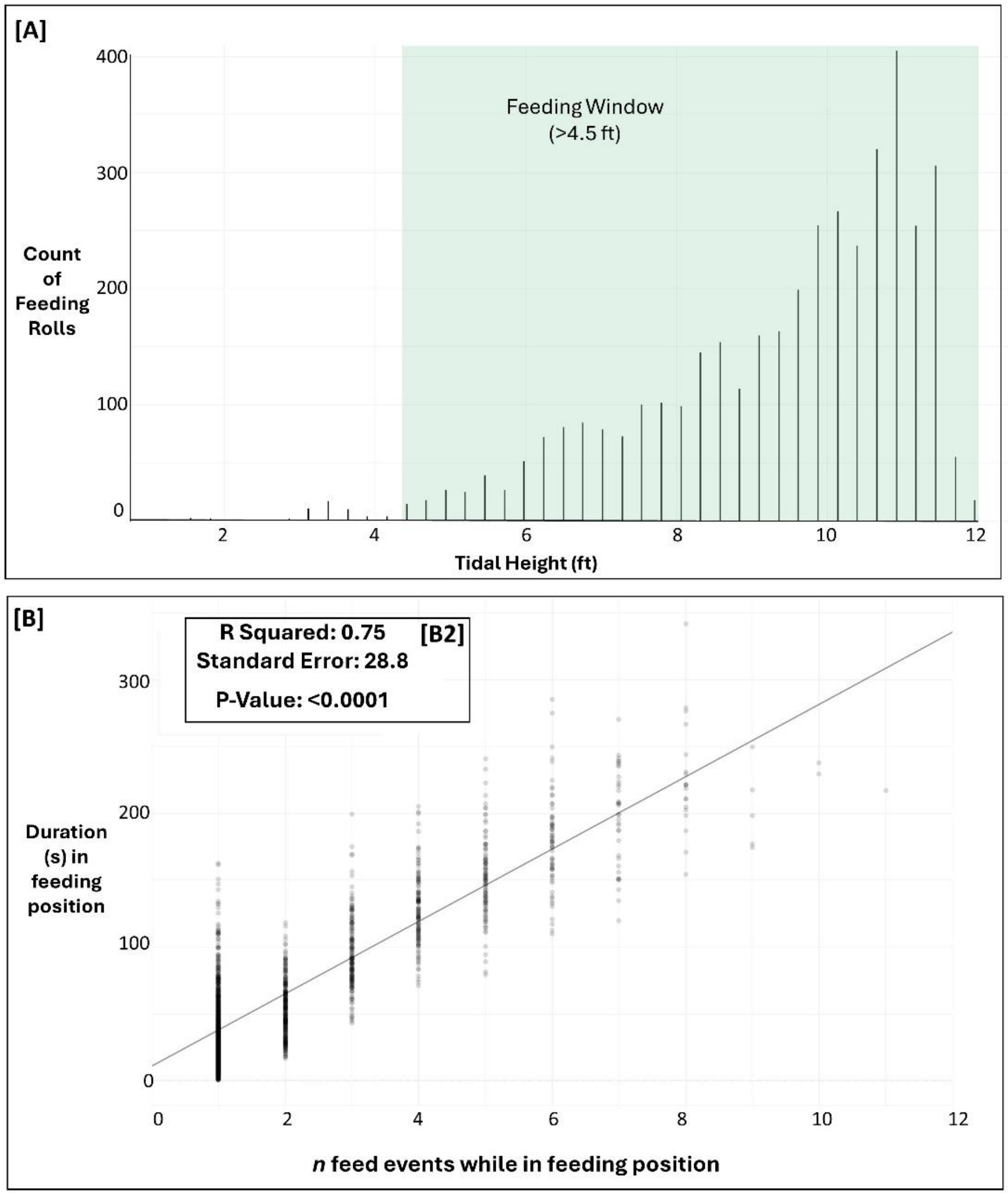

Fig. S1. Methods in describing feeding window and support for feeding event delineation. A) Number of feeding rolls relative to tidal height (ft). The feeding window that resulting feeding rates are calculated from captures over 99% of the distribution of feeding rolls. The feeding window (over 4.5 feet) is highlighted as the green shaded box. B) The distribution of the number of feeding events per feeding roll (x-axis) compared to the time (sec) spent in feeding position for the respective roll (y-axis). Linear regression indicates a strong positive relationship between how long an individual spent, and how many feed events were detected, while in feeding position (B2). Note similarly proportioned ranges across the realm feeding event counts.

Journal of Experimental Biology • Supplementary information

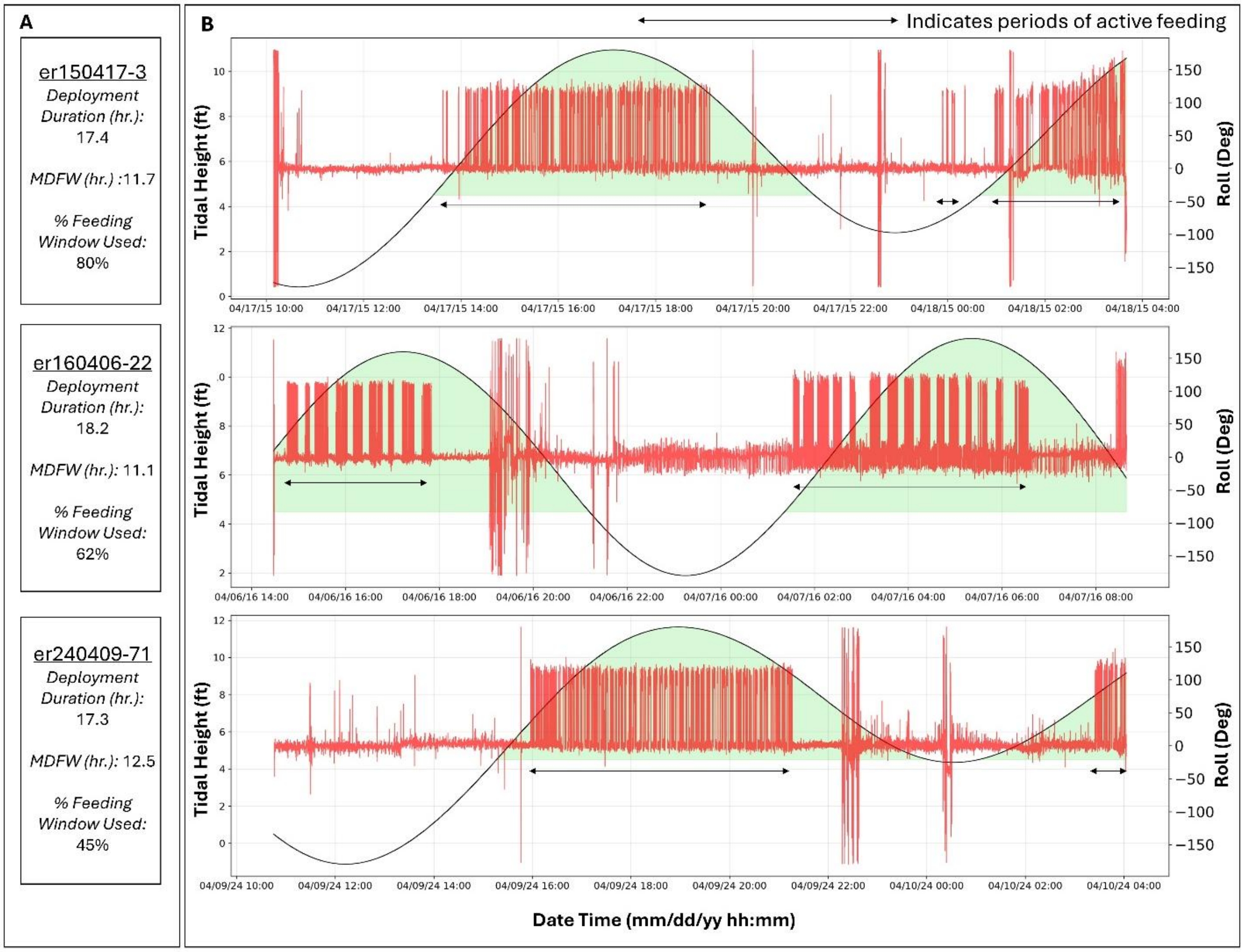

Fig. S2. NPS gray whales may change the proportion of feeding window they utilize in response to differing tidal dynamics. Figure compares the feeding behavior of three unique deployments of similar durations and includes relevant metadata. A) Deployment and feeding metadata associated with each deployment. The deployment ID is underlined and followed by the deployment duration (hours), the mean daily feeding window duration (hours), and the amount (percent) of feeding window this individual was found to be foraging. B) By-deployment plots of respective tidal height in feet (left y-axis; black lines), roll in degrees (right y-axis; red lines), over time (x-axis) in mm/dd/yy HH:mm format. Feeding windows (heights >4.5 feet) are represented as green shaded regions. Periods of active feeding as the horizontal lines with arrows on each subplot. Note that for different deployments, both the low tidal level and the duration of these low tidal periods can vary, potentially leading to changes in foraging effort.

Journal of Experimental Biology • Supplementary information

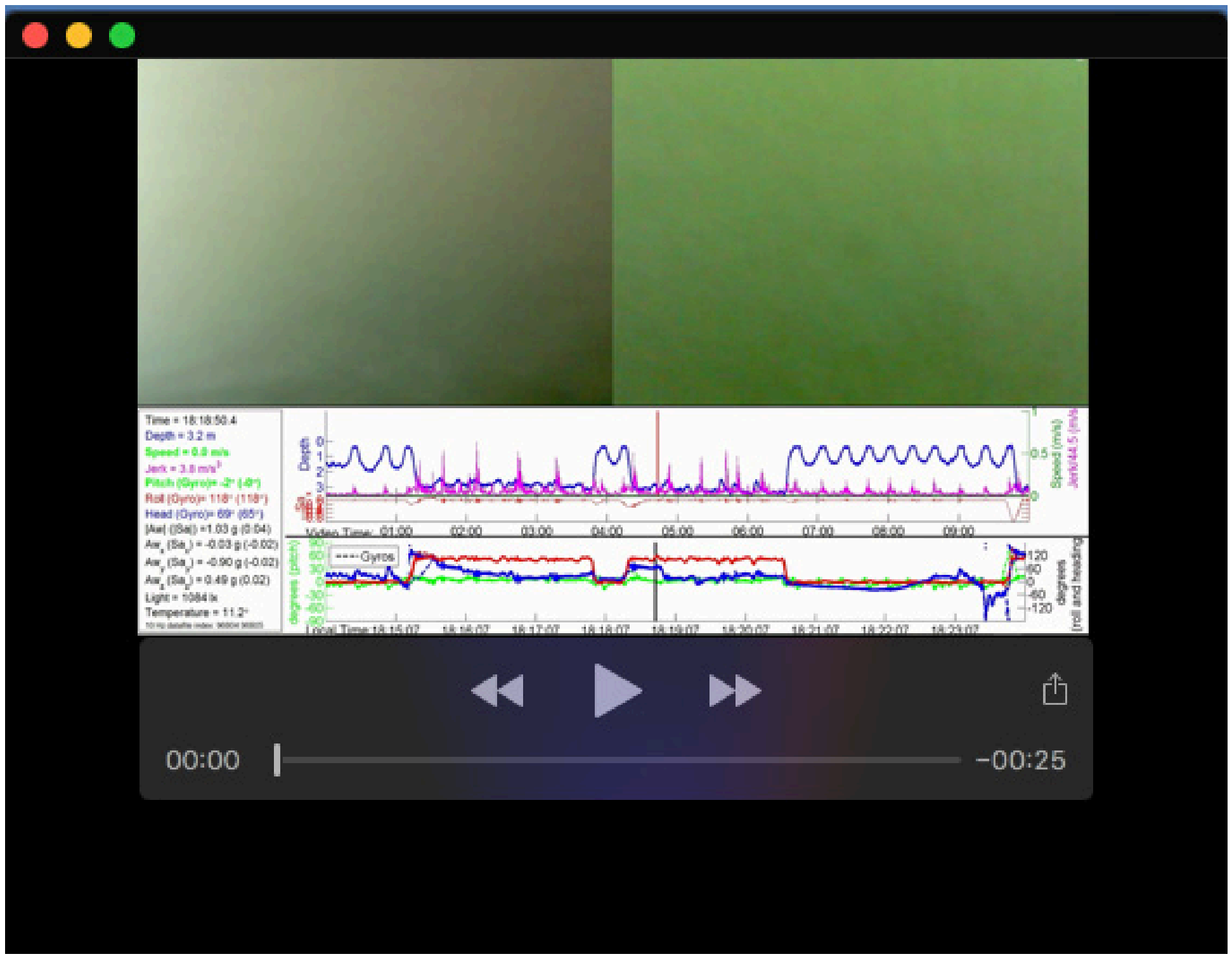

Movie 1. Part one compilation of tag video footage showing observations used to confirm active feeding such as sediment plumes, shrimp burrows, and live shrimp.

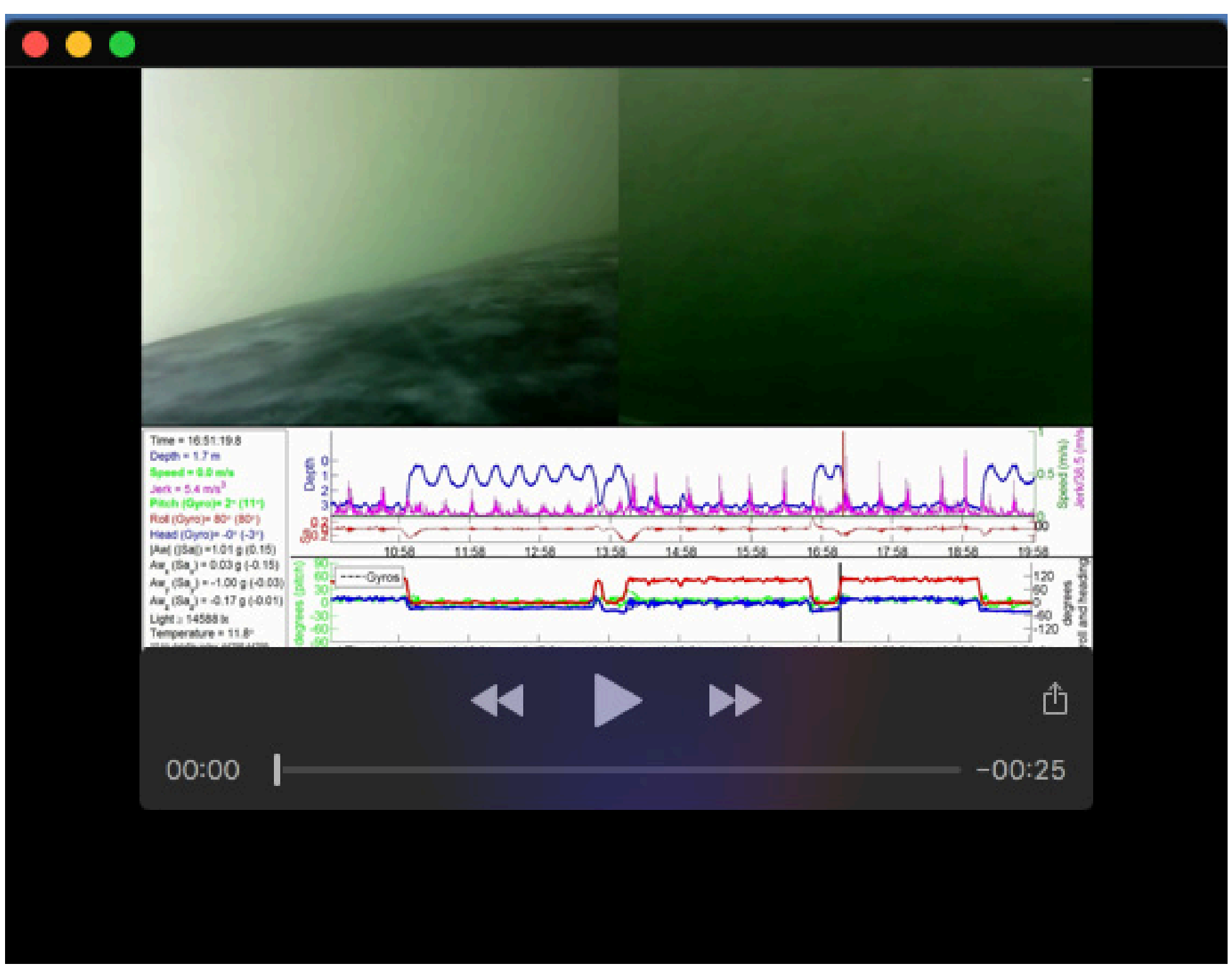

Movie 2. Part two compilation of tag video footage showing observations used to confirm active feeding such as sediment plumes, shrimp burrows, and live shrimp.
